# Mass-selective and ice-free electron cryomicroscopy protein sample preparation via native electrospray ion-beam deposition

**DOI:** 10.1093/pnasnexus/pgac153

**Published:** 2022-08-06

**Authors:** Tim K Esser, Jan Böhning, Paul Fremdling, Mark T Agasid, Adam Costin, Kyle Fort, Albert Konijnenberg, Joshua D Gilbert, Alan Bahm, Alexander Makarov, Carol V Robinson, Justin L P Benesch, Lindsay Baker, Tanmay A M Bharat, Joseph Gault, Stephan Rauschenbach

**Affiliations:** Department of Chemistry, University of Oxford, Mansfield Road, Oxford OX1 3TA, UK; Sir William Dunn School of Pathology, University of Oxford, South Parks Road, Oxford OX1 3RE, UK; Department of Chemistry, University of Oxford, Mansfield Road, Oxford OX1 3TA, UK; Department of Chemistry, University of Oxford, Mansfield Road, Oxford OX1 3TA, UK; Sir William Dunn School of Pathology, University of Oxford, South Parks Road, Oxford OX1 3RE, UK; Thermo Fisher Scientific, Hanna-Kunath-Straße 11, 28199 Bremen, Germany; Thermo Fisher Scientific, Zwaanstraat 31G/H, 5651 CA Eindhoven, The Netherlands; Thermo Fisher Scientific, 5350 NE Dawson Creek Drive, Hillsboro, OR 97124, USA; Thermo Fisher Scientific, 5350 NE Dawson Creek Drive, Hillsboro, OR 97124, USA; Thermo Fisher Scientific, Hanna-Kunath-Straße 11, 28199 Bremen, Germany; Biomolecular Mass Spectrometry and Proteomics, Bijvoet Center for Biomolecular Research and Utrecht Institute for Pharmaceutical Sciences, University of Utrecht, Padualaan 8, 3584 CH Utrecht, The Netherlands; Department of Chemistry, University of Oxford, Mansfield Road, Oxford OX1 3TA, UK; Department of Chemistry, University of Oxford, Mansfield Road, Oxford OX1 3TA, UK; Division of Structural Biology, University of Oxford, Roosevelt Drive, Oxford OX3 7BN, UK; Sir William Dunn School of Pathology, University of Oxford, South Parks Road, Oxford OX1 3RE, UK; Structural Studies Division, MRC Laboratory of Molecular Biology, Francis Crick Avenue, Cambridge CB2 0QH, UK; Department of Chemistry, University of Oxford, Mansfield Road, Oxford OX1 3TA, UK; Department of Chemistry, University of Oxford, Mansfield Road, Oxford OX1 3TA, UK; Max Planck Institute for Solid State Research, Heisenbergstrasse 1, DE-70569 Stuttgart, Germany

**Keywords:** native mass spectrometry, ion-beam deposition, cryo-EM, ice-free sample preparation, structural biology

## Abstract

Despite tremendous advances in sample preparation and classification algorithms for electron cryomicroscopy (cryo-EM) and single-particle analysis (SPA), sample heterogeneity remains a major challenge and can prevent access to high-resolution structures. In addition, optimization of preparation conditions for a given sample can be time-consuming. In the current work, it is demonstrated that native electrospray ion-beam deposition (native ES-IBD) is an alternative, reliable approach for the preparation of extremely high-purity samples, based on mass selection in vacuum. Folded protein ions are generated by native electrospray ionization, separated from other proteins, contaminants, aggregates, and fragments, gently deposited on cryo-EM grids, frozen in liquid nitrogen, and subsequently imaged by cryo-EM. We demonstrate homogeneous coverage of ice-free cryo-EM grids with mass-selected protein complexes. SPA reveals that the complexes remain folded and assembled, but variations in secondary and tertiary structures are currently limiting information in 2D classes and 3D EM density maps. We identify and discuss challenges that need to be addressed to obtain a resolution comparable to that of the established cryo-EM workflow. Our results show the potential of native ES-IBD to increase the scope and throughput of cryo-EM for protein structure determination and provide an essential link between gas-phase and solution-phase protein structures.

Significance Statement Our method creates a direct link between structural information, obtained from cryo-EM imaging, and complementary chemical information, obtained from mass spectrometry. Complementary information from other mass spectrometry-based techniques is increasingly used to accelerate screening of sample conditions, interpret and refine 3D structures, reveal native interactions, and provide information on small ligands and flexible protein regions. However, mass spectrometry and cryo-EM are usually performed separately and correlating the results can be difficult, in particular for heterogeneous samples. In contrast, we demonstrate highly selective sample preparation and unambiguous assignment between mass spectra and structures. Direct imaging after deposition and retention of a dehydrated state offers additional evidence for the close relationship between protein structures in solution and gas phase.

## Introduction

Most structures of biomolecules deposited in the Protein Data Bank (PDB) were obtained by X-ray crystallography, followed by NMR and increasingly electron cryomicroscopy or cryo-electron microscopy (cryo-EM). X-ray crystallography produces high-resolution protein structures from solid, crystalline samples (1), whereas NMR provides information on structure and dynamics of biomolecules in solution (2). Cryo-EM can provide structures even if molecules are intractable to crystallization or too large for NMR, and has recently evolved into a leading method for structural characterization of folded proteins and protein complexes (3,4).

In the conventional cryo-EM workflow, enriched and purified solutions of biological macromolecules are applied to transmission electron microscopy (TEM) grids, which are then blotted, and rapidly submerged into liquid ethane, embedding molecules in thin films of vitreous (amorphous) ice. In general, this method preserves the native structure and conformation (5), prevents dehydration, and retards the spread of radiation damage (6). In single-particle analysis (SPA), high-resolution, 3D EM density maps are reconstructed from low-dose, 2D projection images of thousands to hundreds of thousands single particles (7). Recent technological advances in direct electron detectors, bright electron point sources, energy filters, and aberration-corrected lenses can enable atomic resolution close to 1 Å (7–12). In addition, novel classification algorithms facilitate obtaining multiple structures from heterogeneous cryo-EM samples, and even reveal flexible protein structures (13–15).

Nevertheless, reliable preparation of chemically pure and homogeneous cryo-EM samples remains one of the most important challenges in cryo-EM (16–22). Fragile biomolecules can denature during solution-based enrichment and purification, at the substrate–solvent and air–solvent interface, due to blotting, from evaporation-induced buffer modification, and during plunge freezing (16, 20, 23, 24). As a result, unambiguous classification of single-particle images of different oligomers, conformers, fragments, and contaminants is not always possible, which limits resolution and hides conformational variability and flexibility.

Several methods have been developed to improve cryo-EM sample preparation: Sample heterogeneity can be reduced by cross-linking (25), introduction of stabilizing ligands (26), and sampling of buffers (27). Functionalized substrates or detergents can be used to keep particles away from the air–water or substrate–water interface and reduce preferred particle orientation (28–30). While these methods often allow to circumvent solution-related effects, successful application is not guaranteed for any given biomolecule.

In this study, we present an alternative approach, fundamentally different from conventional cryo-EM sample preparation. We prepare ultra-pure TEM samples in vacuum, by combining native electrospray (ES) with ion-beam deposition (IBD) of *m/z*-filtered protein ions. Key differences in native electrospray ion-beam deposition (native ES-IBD) are as follows: (1) It relies on native ES as source of folded proteins. (2) It replaces solution-based purification and enrichment with continuous accumulation of *m/z*-filtered, gas-phase protein ions in vacuum. (3) Proteins encounter the air–water interface in the electrospray droplet, and are later gently landed on electron-transparent supports in vacuum that are free of ice. We aim to characterize the performance of native ES-IBD of large protein assemblies and evaluate its potential in structural biology.

Native mass spectrometry (native MS) retains proteins in a near-native, folded state, providing information on mass, composition, conformation, and ligand binding states (31). To this end, intact, noncovalently bound protein complexes are transferred from solution into a mass spectrometer using electrospray ionization (ESI) sources at low flow rates, inlet temperatures typically below 100°C, aqueous solvents, and volatile buffers. Because gas-phase protein ions are separated according to their mass-to-charge ratio (*m/z*), native MS can generate ultra-pure gas-phase ion beams. Even proteins from within heterogeneous environments can be distinguished and interrogated. For instance, folded membrane proteins or protein complexes, even with entire segments of the cell membrane, can be transferred into vacuum, enabling purification of proteins or their components within the mass spectrometer (32). In addition, native MS can be coupled with structurally sensitive techniques such as ion-mobility spectrometry (IMS) (33), hydrogen–deuterium exchange (HDX) (34), and various forms of ion activation (35), which provide indirect information on stoichiometry and conformation. In particular, complementary information from native MS can be crucial to guide interpretation and reconstruction of structures from cryo-EM experiments (36–39).

In ES-IBD (also referred to as soft/reactive landing or preparative mass spectrometry), intense, if required *m/z*-filtered, molecular ion beams of defined kinetic energy are deposited onto surfaces in vacuum (40–42). It has been successfully enabling high-resolution imaging of complex molecules by scanning probe microscopy (SPM) (43–48), TEM (49–51), and low-energy electron holography (LEEH) (52, 53). These experiments show that individual peptides, glycans, and proteins can be isolated from a mixture (48), separated from solvent and contaminants, deposited, and imaged. The ES-IBD approach to sample fabrication has been used to address fundamental questions related to gas-phase structures (53), mechanical properties (47, 54), and substrate interactions (55) by direct observation of deformation (47, 54), fragmentation (42, 47), and assembly at surfaces depending on ion-beam composition (45), substrate (46), and landing energy (53, 56).

Protein sample preparation for TEM, based on native MS and IBD, has been suggested previously by Mikhailov et al. (49). In pioneering experiments, they used a modified quadrupole time-of-flight tandem mass spectrometer and negative stain EM and imaged GroEL and apoferritin as globular structures after in-vacuum deposition. However, sample quality was limited by ion-beam intensity below 1 pA, uncontrolled and high landing energy of tens of eV per charge, and the use of staining after landing. Recently, Westphall et al. presented a similar approach, based on a modified mass spectrometer, comprising mass-selective deposition of protein complexes into a thin, liquid glycerol layer, followed by room-temperature negative stain EM. They show reconstruction of near-native protein complex structures after deposition (57). Retaining all previously discussed advantages and challenges caused by the liquid environment, it is closer to the established TEM workflows, though access to high-resolution information is currently limited by the use of negative staining.

For our experiments, we extended and modified a commercial mass spectrometer (Thermo Scientific Q Exactive UHMR instrument) by integrating custom ion-beam deposition hardware and software. Based on this setup, we were able to prepare ice-free native ES-IBD samples of several protein complexes, which were then imaged by cryo-EM. We obtained protein shapes after imaging and 3D reconstruction, consistent with those from vitrified samples, albeit at lower resolution. Without equilibration in solution after landing, we are able to directly probe adsorbed gas-phase structures, which is relevant for the interpretation of results obtained from the broad range of native MS-based techniques (58).

## Results

We have implemented a native ES-IBD instrument based on a commercial, high-resolution, tandem Q Exactive UHMR mass spectrometer, designed for analysis in a mass range up to 80,000 *m/z* (59). We modified the ion-source, instrument operation mode, and added home-made deposition ion-optics and control software (see Fig. S1). The modifications allow for extraction of an ion beam from the collision cell for controlled deposition onto various substrates, including TEM grids, at room temperature and in vacuum. We demonstrate homogeneous coverage of ice-free cryo-EM grids with mass-selected specimens. Ion-beam intensities for mass-selected native proteins range from 10 to 100 pA, and typically allow cryo-EM grids to be prepared within 30 minutes. Careful thermalization and guiding of the ion beam allows to keep landing energies below 2 eV per charge, where covalent bonds are unlikely to be affected (40, 42, 60). The significant improvements of ion-beam intensity and landing energy control, combined with freezing in liquid nitrogen after sample transfer under ambient conditions, and imaging at cryogenic temperatures, enabled us to demonstrate 3D reconstruction after gas-phase purification and gentle surface deposition.

### Native ES-IBD workflow for structure determination

**Fig. 1.**
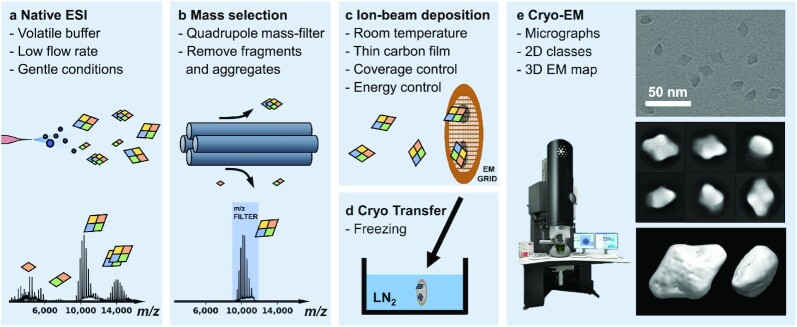
Schematic representation of mass-selective cryo-EM sample preparation using the native ES-IBD workflow. Mass spectra, micrographs, 2D classes, and 3D EM density maps shown here were obtained by applying the workflow to β-gal (compare Fig. 5). (a) Proteins are transferred into the gas phase via native electrospray ionization. (b) The species of interest is mass-selected and separated from fragments, aggregates, and contaminants. (c) The mass-selected ion beam is deposited with defined landing energy on TEM grids. (d) Grids are removed from the deposition chamber and plunged into liquid nitrogen, followed by cryogenic transfer to the microscope. (e) Samples are imaged and micrographs are processed according to established SPA procedures, resulting in 2D classes and 3D EM density maps.

Figure 1 gives an overview of the complete sample preparation workflow from solution to 3D EM density map (see the “Methods” section for details on used materials, protein preparation, native MS, and SPA). Protein solutions are prepared using a standard native MS workflow, including exchange of buffer to volatile ammonium acetate (61). To achieve the highest *m/z* precision, ions are usually desolvated using collisional activation in the atmosphere-to-vacuum interface, sometimes followed by additional activation in the quadrupole collision cell. Instead, for all experiments presented here, we use reduced electric potential gradients throughout the instrument to promote soft, nonactivating conditions, while retaining high ion transmission.

**Fig. 2.**
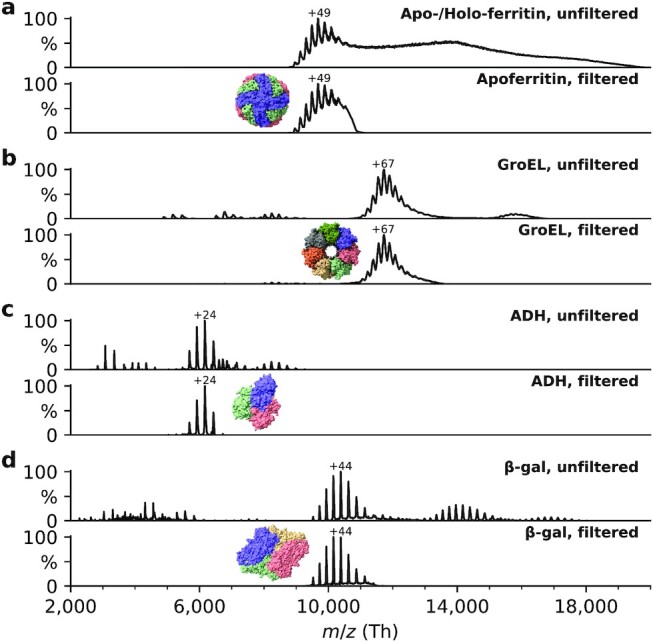
Nonactivating, native mass spectra of apo/holo-ferritin, GroEL, ADH, and β-gal. For native ES-IBD experiments, native oligomers were mass selected, i.e. tetramer for ADH and β-gal, tetradecamer for GroEL, and 24-mer for apoferritin. Holoferritin (right of upper panel in (a)) shows no resolved charge states, due to the continuous size distribution of iron cores in our sample. For all other proteins, the most abundant charge states are labeled. They are much lower than typical charge states from denatured samples indicating folded gas-phase structures.

Initially, mass spectra are acquired to ensure native ionization conditions, evaluate nano-electrospray stability, and select a quadrupole mass filter transmission range to separate the protein or protein complex of interest from other components in the solution. Figure 2 shows corresponding unfiltered and filtered mass spectra for a range of protein assemblies, including apo/holo-ferritin, alcohol dehydrogenase (ADH), β-galactosidase (β-gal), and GroEL. These molecules cover a mass range from 147 (ADH) to 803 kDa (GroEL), and have characteristic shapes, and high-quality models are available in the PDB.

The instrument mode is then switched to guide the mass-selected ion beam through the collision cell, where the ions are thermalized before entering a custom deposition stage and reaching a custom, room-temperature sample holder at a pressure of 10^−6^ mbar. An integrated, retarding-grid ion-current detector records the ion-beam intensity, total beam energy, and beam-energy distribution. The concentration of the protein solution and ion-source parameters are optimized for beam intensity and stability. The total beam energy (kinetic energy and potential energy relative to ground) typically decreases by only 1.5 eV per charge between the collision cell and sample, due to collisions with the background gas, indicating gentle, nonactivating transfer through the custom DC ion optics. The full width at half-maximum (FWHM) of the beam energy distribution is typically below 1 eV per charge.

Prior to deposition, TEM grids with carbon films are placed into the sample holder, and inserted into the deposition chamber via a vacuum load-lock. Plasma-cleaning to produce a more hydrophilic surface is not required, as our method bypasses the application of a liquid to the grid at any point. The deposition current on the grids is monitored using a picoammeter to align the ion beam and obtain the total deposited charge, in picoampere hours (pAh), by integrating the deposition current. Typically, 10 to 15 pAh is collected in 30 minutes. A charge of 1 pAh corresponds to 22.5 × 10^10^ elementary charges, the charge deposited from a 1 pA ion-beam over 1 hour. With an average charge state of 45, such as for β-gal, 15 pAh correspond to 7.5 × 10^9^ particles.

Particle density measurements across the grid typically indicate a Gaussian-shaped particle density distribution with an FWHM around 1 mm, meaning that the entire grid is covered without moving the beam and the particle density increases towards the grid center (59).

After deposition, TEM grids are retrieved via the vacuum load-lock, transferred under ambient conditions, and manually plunged into liquid nitrogen, instead of liquid ethane, within 2 minutes. Note that fast cooling rates as used for vitrification are not required due to the absence of water. Samples are then stored in liquid nitrogen. Subsequent sample transfer, imaging, and data processing proceeds using standard SPA workflows established for samples with vitrified ice.

### Preparation of clean and homogeneous samples of controlled density

**Fig. 3.**
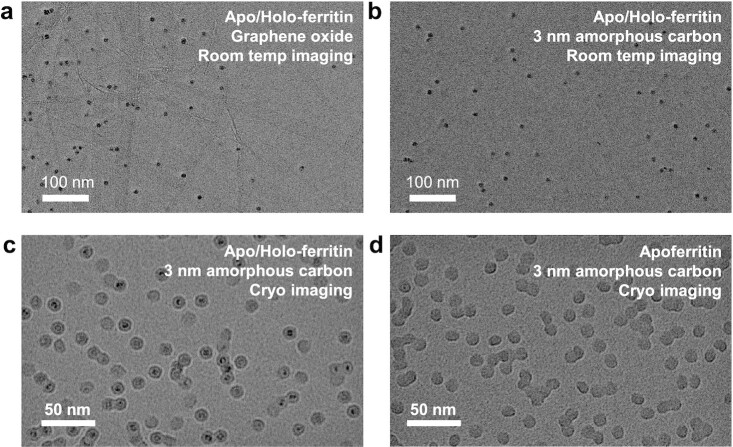
(a) Room-temperature TEM micrograph of ferritin, deposited on a home-made graphene oxide film. Particles are found mainly in areas with thick GO film (left side). (b) Room-temperature TEM micrograph of ferritin on a 3 nm amorphous carbon film under identical deposition and imaging conditions, demonstrating a clean sample with homogeneous particle density. (c) Cryo-EM micrograph of an unselective ice-free native ES-IBD sample of apo/holo-ferritin, corresponding to the mass spectrum shown in Fig. 2(a) (top). (d) Cryo-EM micrograph of a selective apoferritin sample, corresponding to the mass spectrum shown in Fig. 2(a) (bottom). Both samples were prepared from the same solution.

Initial experiments were conducted with deposition and imaging at room temperature, i.e. without freezing. We deposited an apo/holo-ferritin mixture on Quantifoil grids with home-made graphene oxide (GO) films. The high contrast of the iron core of holoferritin allowed investigation of particle density and distribution at room temperature, even though the protein was hardly visible. Figure 3(a) shows a variation of particle density with GO substrate film thickness across a single micrograph. The particle density is high and homogeneous on thick GO films (left), but only few particles are found on thin films (right). We rationalize this observation by suppression of thermal particle diffusion due to stronger Van der Waals interaction and higher density of edges and defect sites on the thicker areas, as previously observed for the adsorption of clusters on freestanding graphene (62).

Thus, to maximize contrast by using ultra-clean and thin films like weakly interacting GO or monolayer graphene, thermal diffusion has to be suppressed by surface modification or cooling. Instead, here we proceeded to use commercial 3-nm-thick amorphous carbon films, which suppress thermal diffusion and provide a consistent homogeneous background in EM micrographs over the entire grid. This enabled us to obtain homogeneous coverage as shown in Fig. 3(b). The majority of particles are well separated from each other rather than aggregated. The entire grid is covered with increasing particle density towards the center, allowing to select an ample number of grid squares with ideal density for data collection.

We then added a manual freezing step in liquid nitrogen directly after deposition and transfer under ambient conditions. Cryogenic temperatures were maintained throughout the transfer to the microscope and imaging. Figure 3(c) and (d) shows samples prepared by depositing an apo/holo-ferritin mixture and mass-selected apoferritin, respectively. In contrast to the room-temperature micrographs, the ferritin protein shells are clearly visible around the iron cores when imaging samples at cryogenic temperatures after deposition. Apoferritin, barely visible when imaging at room temperature, can be clearly identified as well. The absence of iron cores in Fig. 3(d) demonstrates the power of selective sample preparation.

### Protein shape is retained after electrospray, gas-phase purification, and deposition

**Fig. 4.**
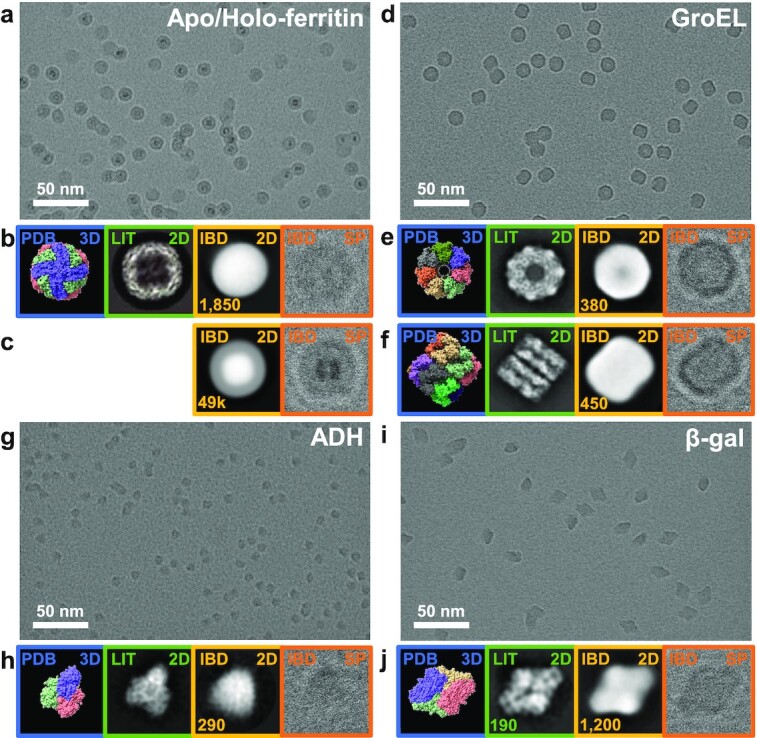
Unfiltered cryo-EM micrographs of ice-free native ES-IBD samples: (a) apo/holo-ferritin, (d) GroEL, (g) ADH, and (i) β-gal. Panels beneath the micrographs show, from left to right, 3D models from the PDB (blue, PDB 3D), 2D classes from plunge-frozen cryo-EM samples from the literature (green, LIT 2D), and 2D classes (yellow, IBD 2D) and representative single particles (orange, IBD SP) from ice-free native ES-IBD samples. Panels (b) and (c) show apo- and holo-ferritin, panels (e) and (f) show GroEL top and side views, and panels (h) and (j) show ADH and β-gal. Three dimensional PDB models were rendered with ChimeraX (67) using PDB entries 7A6A (apoferritin), 5W0S (GroEL), 7KCQ (ADH), and 6CVM (β-gal). Two dimensional classes for apoferritin, GroEL, ADH, and β-gal were taken from literature (12, 63, 64) and the RELION 3.0 tutorial data set. The number of particles in the 2D classes is given where available. Note that panel (a) is reproduced from Fig. 3(c) for completeness.

We applied the same workflow to prepare and image native ES-IBD samples for GroEL, ADH, and β-gal. For each sample, we collected 50 micrographs, resulting in up to 3,000 single particles. We performed 2D classification in RELION 3.1 (see the “Methods” section). The resulting micrographs are shown in Fig. 4, along with 2D classes and representative single particles as well as corresponding 3D models from the PDB and published 2D classes (12,63, 64) obtained from traditional plunge-frozen cryo-EM samples of proteins in vitrified ice from grids with holey carbon films.

The single apoferritin particle in Fig. 4(b) appears circular, but the corresponding 2D class has a slightly oval shape, suggesting a deformation of the protein shell during the workflow. For holoferritin, Fig. 4(c), the single-particle shell is round on the outside and rectangular on the inside, closely resembling the PDB structure shown for apoferritin. This is confirmed in the 2D class, but further analysis is inhibited as the classification algorithm is confounded by the high-contrast iron core. Interestingly, there are no indications of a localized deformation of the protein shell as for apoferritin. This result is consistent with data from IMS, which suggests a gas-phase collapse of the apoferritin cavity and stabilization of holoferritin by the iron core (65, 66).

Figure 4(d) shows a micrograph of a GroEL sample. Very high contrast is observed due to the high mass of 803 kDa and absence of ice. Top and side views can be identified unambiguously. The 2D classes of top and side views clearly show features of the characteristic barrel shape that are already apparent in the single-particle images, including the central cavity, a heptameric symmetry in the top view, and the notch between the two heptamer rings in the side view. Further substructure, as present in the literature 2D classes, is not visible.

A micrograph of a sample of ADH, at 147 kDa the lowest mass protein complex in our study, is shown in Fig. 4(g). It demonstrates the remarkable contrast of ice-free samples, in which shapes can be recognized in unfiltered micrographs, even for smaller proteins. Individual particles show characteristic triangular shapes which become much clearer in the 2D class shown in Fig. 4(h). However, due to a lack of homogeneous internal structure in the single-particle images, specific orientations could not be assigned unambiguously.

Finally, Fig. 4(i) shows a micrograph of a sample of β-gal, which is commonly used as a standard in cryo-EM due to its relatively high mass (466 kDa), high stability, and characteristic shape. In contrast to apo/holo-ferritin and GroEL, β-gal has no iron core or cavity. Again, individual particles and their orientations can be identified unambiguously from the unfiltered micrograph. The most prominent diamond-shaped 2D class and a corresponding single particle are shown in Fig. 4(j).

Generally, there is no evidence for unfolding into extended polypeptide chains or fragmentation into subunits. The significantly improved contrast and well-defined shape in the 2D classes of the ice-free samples indicate consistency of low-resolution structural features among particles and preservation of quaternary structure. Particle dimensions in 2D classes indicate no lateral deviation from literature values. However, there is a consistent lack of defined internal structure compared to the 2D classes from conventionally prepared samples with vitreous ice.

### Three dimensional reconstruction based on native ES-IBD samples

To assess the currently accessible structural information in more detail, we collected approximately 3,000 movies of β-gal from which we obtained 50,000 single-particles images, a significantly larger data set than above. For direct comparison, we prepared a control sample by applying the solution used for native ES-IBD directly to a grid with holey carbon film, followed by conventional blotting and plunge freezing. Approximately 900 movies were recorded with identical microscope settings (see the “Methods” section), resulting in 90,000 particles.

**Fig. 5.**
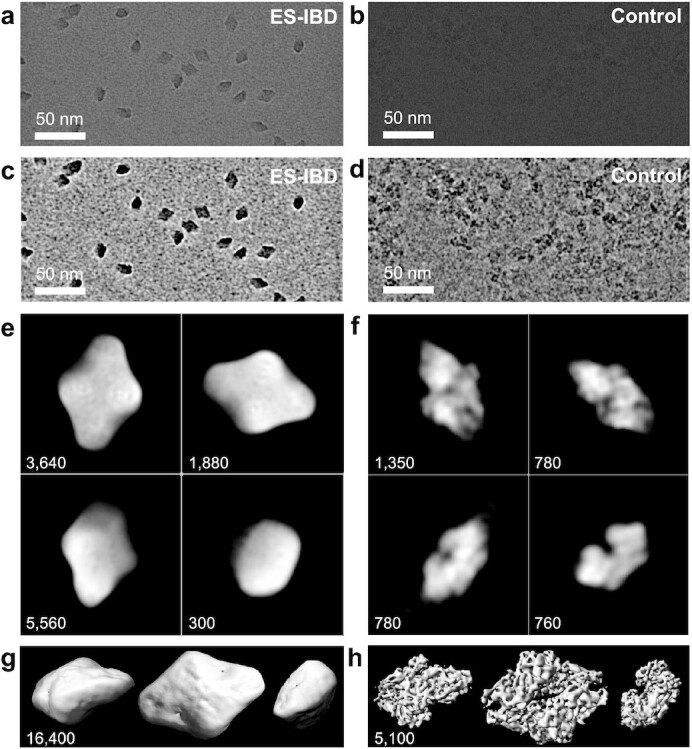
Unfiltered EM micrographs (a and b), EM micrographs band-pass filtered between 10 and 200 Å (c and d), representative 2D classes (e and f), and 3D EM density maps (g and h), for a native ES-IBD and a plunge frozen control sample of β-gal, respectively. The number of particles in the 2D classes and 3D maps is indicated in the panels.

Figure 5 shows unfiltered EM micrographs, band-pass filtered EM micrographs, 2D classes, and 3D EM density maps of both samples. As described above, the particle contrast for the native ES-IBD sample is very high, the particle density is very homogeneous, and individual particles are well separated from each other. In contrast, the unfiltered micrograph of the vitrified control sample shows aggregated particles. Individual particles and orientations are hard to identify by eye and only become visible after band-pass filtering. After 2D classification, randomly distributed particle orientations, shown in Fig. 5(f), are obtained for the control sample, whereas a strong preferred orientation is observed for the native ES-IBD sample. The latter exclusively shows three orientations, shown in Fig. 5(e), with a particle percentage of 56% (diamond-shaped), 43% (tilted), and <1% (top).

Two dimensional classes of the control sample show features of strong internal contrast, corresponding to secondary structure. These features are missing in the native ES-IBD sample, despite a much better contrast relative to the background. However, a characteristic symmetric pattern can be observed in the diamond-shaped 2D classes (see Fig. S2 for marked features). It consists of clusters of four high-density spots, and four low-density spots around the center as well as at both tips. The smallest distance between the high-density spots is 8 Å. In addition, two of the opposing diamond edges have lower contrast than the other two. The same pattern is observed when plunging into liquid ethane instead of liquid nitrogen (see Fig. S2).

Next, we look at the 3D EM density maps of the ES-IBD and control sample in Fig. 5(g) and (h), respectively. The level of information is consistent with that in the 2D classes. For the control sample, a 9 Å (0.143 gold-standard FSC) 3D EM density map was obtained, which shows very good agreement with the secondary structure from the PDB (see Fig. S3). The corresponding 3D map from the native ES-IBD sample reliably reproduces the external features but lacks internal detail (see Fig. S4 and Movie 1 in the “Supplemenatry Material” section).

## Discussion

By showing data from five different proteins prepared via native ES-IBD, we demonstrate reproducibility of our workflow within the current limits of sample quality. Our results clearly show that proteins remain folded and subunits in protein complexes remain attached to each other in their native orientation during sample preparation using native ES-IBD, despite dehydration, electrostatic repulsion within charged protein ions, collision with the substrate, and prolonged exposure to the substrate–vacuum interface at room temperature. For β-gal, subnanometer resolution is routinely achieved using conventional vitrified samples, but this is not currently attained with native ES-IBD samples. In the following, we discuss the main differences between the two sample preparation methods that may explain this discrepancy.

### Solution and imaging

The successful generation of a subnanometer 3D map for the control sample shows that access to secondary structure is neither limited by the preparation of the solution for mass spectrometry, the microscope performance, and settings, nor limited by the number of collected single-particle images.

### Dehydration

Simulations suggest that a 3-Å-thick hydration shell, equivalent one to two molecular layers of water, can be sufficient to conserve the bulk water protein structure in the gas phase (68). For β-gal, this would correspond to a few thousand water molecules, which is comparable to the 4,194 structural water molecules that are located mainly at the surface of the cryo-EM based PDB model (69). As simulations show that water tends to cluster in crevices on the protein surface (70), the actual number of molecules required to cover the entire surface will be notably higher.

For all systems tested here, less than 200 water molecules remained attached to the gas-phase protein ions (see the “Methods” section), far below the number for a full solvation shell. This removal of the majority of structural water molecules leads to formation of intramolecular hydrogen bonds and reduced screening of electrostatic interactions. MD simulations and gas-phase experiments indicate that dehydration can lead to a range of kinetically trapped gas-phase structures; however, the native secondary structure is typically preserved to a large extent and gas-phase proteins are able to refold, at least partially, upon rehydration (68, 70–73). In particular, it has been shown that viruses and monomeric enzymes can retain their biological activity after ion-beam deposition onto liquids or dry surfaces (55, 74, 75). Recently, with the instrument used in this study, we have demonstrated activity of the tetrameric protein complex ADH after deposition onto a dry surface in vacuum followed by subsequent rehydration (59). Structural rearrangements of gas-phase proteins following dehydration in vacuum occur on a timescale on the order of pico- to milliseconds (76). As we expect the deposited proteins to complete any structural changes on a similar time scale, variations in the much longer deposition time should not be relevant.

In ESI, the lifetime of the hydration shell can be extended, for example by background-gas cooling in the source and use of an ESI buffer solution with low vapor pressure and high evaporative cooling rate. It is thus conceivable to find conditions where proteins that retained native structures throughout the mass spectrometer, due to a sufficiently large hydration shell, can be deposited. This approach comes at the cost of reduced *m/z* selectivity due to convolution of mass spectra. Instead, controlled rehydration after mass selection, compatible with cryo-EM, should be feasible given sufficient control over local temperature and water partial pressure. In both cases, cryogenic cooling of the sample is required to maintain hydration during grid transfer and storage. Note that in our current workflow, particles can, in principle, rehydrate during the brief transfer at ambient conditions. The limited control over rehydration during this step prevents separation of its effect on the molecular structure from that of the other discussed factors.

### Landing

Particles may be deformed by the collision with the carbon film. In the present case, β-gal molecules land with a kinetic energy of 2 eV per charge, corresponding to a velocity of about 200 m/s. The nature and severity of deformation caused by this type of collision are currently poorly characterized. Previous studies show that covalent bonds are unlikely to be broken (40,42, 60), but conformation can change (54). Localized deformation of the protein surface and random orientations on impact could introduce sample heterogeneity that leads to loss of information during averaging. It is intuitive to expect a larger deformation when a protein lands on a sharp edge rather than a flat plane. Indeed, when using all particles and allowing for asymmetry in the 3D reconstruction for the ice-free native ES-IBD sample, we obtained classes that show a localized indentation close to one of the narrow tips of β-gal (see Fig. S5 in the “Supplemenatry Material” section).

### Substrate

The protein samples spend between 2 and 30 minutes at the highly asymmetric substrate–vacuum interface at room temperature before being frozen. As we do not see notable variations of quality across single particles and 2D classes, we expect surface-induced structure changes to occur on a much shorter time scale, similar to dehydration. The low-contrast edges in the diamond-shaped class of β-gal (see Fig. S2c) possibly indicate a stronger deformation of the two subunits that are in direct contact with the substrate. The effect of the substrate–vacuum interface on particle orientations and structural integrity has been characterized for a broad range of materials (40). Its effect on proteins, however, is still poorly understood and challenging to control.

### Preferred orientation

Proteins in the ice-free native ES-IBD samples show strong preferred orientation. We rationalize this with a maximization of surface contact area driven by the formation of noncovalent bonds with the surface in the absence of a solvent environment. Preferred orientation can be a limiting factor for 3D reconstruction. However, given that we see reduced detail already in 2D classes, we conclude that preferred orientation is not currently the main limiting factor. To reduce the effects of preferred orientation, additional SPA data could be acquired on a tilted sample (77). In addition, electron cryotomography and subtomogram averaging (STA) can be used to reduce missing regions in Fourier space in the final 3D reconstruction (78).

### Radiation damage

While the dependence of radiation damage on temperature has been investigated thoroughly (79), there is little research on the role of the ice layer, as direct comparison with ice-free samples was previously not possible. Figure S6 shows 2D classes obtained from the same set of particles at different doses for a single class of the native ES-IBD and control sample, respectively. For the control sample, the particle contrast in low-dose data were not sufficient for successful particle alignment into a 2D class. In comparison, the particle contrast in low-dose data for the ES-IBD sample was sufficient. This suggests that lower doses can be used for ES-IBD samples due to enhanced contrast, which can, in principle, reduce radiation damage effects in future applications. However, the limited resolution does not allow us to quantify the effect of radiation damage, or to separate it from other discussed factors.

### Beam-induced motion

While we have discussed substrate-dependent thermal diffusion of proteins at room temperature, the substrate dependence of beam-induced motion on ice-free samples under cryo conditions remains to be characterized. For β-gal, the measured beam-induced motion on the sample prepared by native ES-IBD was only half of that on the vitreous control sample, as shown in Fig. S7, and is thus not a limiting factor here. The difference seems plausible given that tension in the ice is a main contributor to beam-induced motion (80).

### SPA

It is worth considering that data processing in the SPA workflow is highly optimized for vitrified samples and may require adjustment to obtain more information from ice-free native ES-IBD samples.

We conclude that the overall shape of protein assemblies and with it the secondary structure is largely preserved, but we introduce heterogeneity in the secondary and tertiary structures, by dehydration, landing, or surface interactions, which limits the amount of information that can be obtained by averaging techniques. A thorough characterization of the individual aspects discussed above is needed to improve sample quality and thus resolution.

## Conclusions

We have implemented native ES-IBD as a novel approach for reproducible preparation of ice-free, homogeneously covered cryo-EM samples of controlled density. This method provides sample purity by mass-selecting a native protein ion-beam in a mass spectrometer. The extensive optimization of the solution-based purification and vitrification is replaced by optimization of native MS conditions following established protocols, which typically requires less expensive instrumentation, provides faster feedback, and offers orders of magnitude higher selectivity.

With our prototype instrument, we demonstrated the fabrication of cryo-EM samples for a diverse selection of protein assemblies (apo/holo-ferritin, GroEL, ADH, and β-gal). Particles stand out clearly against the background, even for proteins with a molecular weight around 150 kDa, and different orientations can typically be distinguished by eye in unfiltered micrographs.

Protein conformation is largely preserved during transfer onto a TEM grid by carefully controlled native MS and soft landing. Further, we showed that low-resolution 3D reconstruction from ice-free samples is possible using SPA. The obtained protein shapes match expectations from the literature, but a markedly lower spatial resolution can be understood by small, random changes in the secondary and tertiary structures of each protein, due to exposure to the electrospray process, vacuum, landing, or adsorption.

After 40 years in which the underlying principles of cryo-EM specimen preparation remained essentially unchanged (18, 22), our results show the potential of native ES-IBD to enable a variety of truly novel workflows for structure determination that can contribute, for example, to a better understanding of pathological processes and faster drug development. Establishing cryo-EM with samples from mass-selected protein deposition provides a great variety of research opportunities for structural biology, because it directly relates chemical information to biological structure.

## Outlook

Native ES-IBD is in an early stage of development and many challenges have to be addressed to approach the resolution of established cryo-EM workflows. However, the mass-spectrometric information, selectivity, and strong contrast even in the unfiltered micrographs can be useful for screening and interpretation of higher resolution structures obtained using conventional cryo-EM. Further, native ES-IBD can provide complementary information to help address the challenges of the plunge freezing workflow discussed in the Introduction. There may be great potential in eliminating solvent and ice-related effects, including denaturation at the air–solvent interface, strong and inhomogeneous background signal, unintentional devitrification, beam-induced motion of the ice, and inhomogeneous particle distribution.

To increase sample quality, characterization of the effects of various substrates and surface modifications on thermal motion, sample stability, and preferred orientation is required. In particular, direct deposition onto cryogenically cooled grids, in combination with thin ice layers or inert-gas matrices (81), may reduce preferred orientation and help to reduce deformation during landing and exposure to the electron beam. Maintaining hydration, achieving controlled rehydration, or landing in a liquid environment, the latter recently demonstrated by Westphall et al. (57), has the potential to preserve or restore protein structures closer to those in the native environment. These steps require significant modifications to the experiment to achieve a high level of control of grid temperature and protective layers during and after deposition.

Alternatively, real single-particle techniques like LEEH might ultimately provide access to the structure of dehydrated proteins, independent of the structure variations in particle ensembles. As the secondary structure should be largely retained, these dehydrated structures may then be rehydrated computationally to approach native hydrated structures (68).

We expect that landing experiments employing liquid layers and solids will contribute to a better understanding of the relation between native solution-phase and native-like gas-phase structures of biomolecules. For example, the question to what degree native structure is retained during or restored after gas-phase transfer and deposition can be answered using a combination of such approaches.

Imaging proteins after controlled gas-phase exposure allows to address fundamental questions on the nature of the native-like gas-phase state, in particular conformational changes and side chain collapse, widely debated in native MS and IMS (66, 82, 83). The interaction of a protein with different solid surfaces in a hyperthermal collision is of great interest, as it provides a direct means to probe mechanical properties of the protein. While we typically perform experiments in the soft-landing regime to maintain biochemically relevant stoichiometry and native conformation, activation can be readily achieved, either within the mass spectrometer or by reactive landing or surface induced dissociation, to image collision-energy specific states and fragments (42, 54).

Significant improvements in ion transmission are possible (84) and promise fast preparation of ultra-pure samples of individual complexes, charge states, and even ligand bound states. Collection of sufficient particles for image reconstruction on a chromatographic timescale also appears possible. Due to the modular design of our deposition stage, the workflow can be modified to allow preparation of conformation-selective samples using IMS. This would also enable purification of species that cannot be separated by a quadrupole mass filter because they are too close to each other or even overlapping in *m/z*. As we based our workflow on a commercial instrument, all these abilities can be transferred to other labs with reasonable effort.

## Methods

### Protein preparation

Soluble proteins, alcohol dehydrogenase (ADH, A7011-15KU), β-gal (G3153-5MG), ferritin (F4503-25MG), and GroEL (chaperonin 60, C7688-1MG) were purchased from Sigma-Aldrich and used without further purification unless otherwise specified. Ammonium acetate (7.5 M, A2706-100ML), MeOH (1060352500), acetone (1000145000), and buffer components for the reconstitution of GroEL, Tris (93362-250G), KCl (P9541-500G), EDTA (DS-100G), MgCl_2_ (63068-250G), and ATP (A6419-1G) were also purchased from Sigma-Aldrich. All concentrations are calculated with respect to the most abundant oligomers. Lyophilized powders of alcohol dehydrogenase and β-gal were resuspended in 200 mM ammonium acetate (pH 6.9) to a final concentration of  50 µM. The saline ferritin stock solution had a concentration of 260 µM.

GroEL was reconstituted from lyophilized powder. To this end, the powder was dissolved in 100 µL MeOH and 400 µL buffer, containing 20 mM Tris, 50 mM KCl, 0.5 mM EDTA, 5 mM MgCl_2_, and 0.5 mg/mL ATP, mixed for 2 hours at room temperature, precipitated by adding 500 µL cold acetone, and centrifuged to form a pellet. After disposing of the supernatant, the pellet was resuspended in 250 µL of the original buffer and gently mixed overnight. The final solution had a concentration of  5 µM and was aliquoted and stored at −80°C until use.

All proteins were desalted by passing through two P6 buffer exchange columns (7326222, Bio-Rad), equilibrated with 200 mM ammonium acetate (pH 6.9). If applicable, they were then diluted in 200 mM ammonium acetate (pH 6.9) to reach the concentration used for native MS: 5 µM (ADH), 10 µM (β-gal), 8 µM (ferritin), and 5 µM (GroEL). Buffer exchange was always done on the day of deposition.

### Native mass spectrometry

Borosilicate glass capillaries (30-0042, Harvard Bioscience) were pulled into nano-electrospray ionization emitters, using a pipette puller (P-1000, Sutter Instrument), and gold coated using a sputter coater (108A/SE, Cressington). For native MS, up to 10 µL of protein solutions was loaded into an emitter and the emitter tip was clipped to produce an opening of 0.1 to 10 µM (85). Electrospray ionization was initiated by applying a potential of 1.2 kV and a gentle nanoflow pressure (<200 mbar above atm). Modifications of the ion source are described in Fig. S1.

General instrument conditions were as follows: source DC offset = 21 V, S-lens RF level = 200 (300 Vp-p), transfer capillary temperature = 60°C, ion transmission settings set to “high *m/z*” (700 Vp-p for the injection flatapole, and 900 Vp-p for the bent flatapole, transfer multipole, and collision cell), detector optimization “high *m/z*,” injection flatapole = 5 V, interflatapole lens = 4 V, bent flatapole = 2 V, transfer multipole = 0 V, collision-cell pressure setting = 7 (N_2_), collision-cell multipole DC = −5 V, and collision-cell exit lens = −15 V. For collection of mass spectra, the instrument was operated in standard mode (86), and for ion deposition, a modified scan matrix was used that allowed ions to pass through the C-trap and collision cell directly into the deposition stage, without trapping. The number of water molecules attached to the protein complex ions under those conditions was estimated to be less than 200, by comparison to mass spectra after desolvation in the collision cell. The mass difference may also be explained by the presence of residual salt.

### TEM grid preparation

Copper TEM grids with mesh size 400 were purchased from Agar Scientific, including 3 nm amorphous carbon on a lacey carbon film (AGS187-4) and holey carbon film (AGS174-3). A graphene oxide layer was added to the latter by plasma cleaning for 5 minutes, drop casting of 3 µL graphene oxide suspension (763705-25ML, Sigma-Aldrich), diluted in water to 0.2 mg/mL, blotting with filter paper (11547873, Fisherbrand) after 1 minute, followed by three washing and blotting steps with water. No further treatment was applied to grids before deposition.

The control sample for β-gal was prepared using ammonium acetate solutions used for native ES-IBD and a Cu 200 mesh grid (Q2100CR2, Quantifoil) with 2 µm holes and 2 µm spacing between the holes. Three microliters of a 5 µM solution was applied to the grid, followed by blotting and plunging into liquid ethane, using a Vitrobot (Thermo Fisher Scientific) at a relative humidity of 100% and a temperature of 10°C.

### Image acquisition and processing

All micrographs were collected using microscopes at the COSMIC Cryo-EM Facility at South Parks Road, University of Oxford, UK. Room-temperature screening was done on a Talos F200C (Thermo Fisher Scientific) cryo-TEM equipped with a Ceta 16M CMOS camera. Micrographs of native ES-IBD samples of apo/holo-ferritin were imaged on a Titan Krios 300 kV cryo-TEM (Thermo Fisher Scientific) equipped with a BioQuantum energy filter operated at a slit width of 20 eV and a K3 direct electron detector (both Gatan). All other data were acquired on a Talos Arctica 200 kV cryo-TEM with a Falcon 4 direct electron detector (both Thermo Fisher Scientific). Manual and automated data acquisitions were controlled using EPU software (Thermo Fisher Scientific).

All micrographs were recorded using a range of defocus settings between −1 and −3 µm. For the unfiltered micrographs shown in this work, the color range was adjusted to the data range, but no data were cut off and no nonlinear adjustments were applied. Typically, 50 micrographs were recorded per sample giving up to 3,000 particles and the magnification was 180E3 corresponding to a pixel size of 0.78 Å.

For β-gal, two larger data sets of 3,000 and 900 EER (87) movies were collected for the native ES-IBD and control sample, providing 50,000 and 90,000 particles, respectively. The magnification for this collection was 240E3 corresponding to a pixel size of 0.59 Å and the total exposure was 40 *e*/Å^2^.

For SPA, data were processed using RELION 3.1 (88). Motion-corrected MRC files were generated from EER files, using RELION’s own implementation of the MotionCor2 algorithm (89). Contrast transfer functions (CTFs) were estimated using CTFFIND 4.1 (90) and used for CTF correction in the following steps. The high contrast obtained for native ES-IBD samples allowed for reliable automated particle picking based on a Laplacian-of-Gaussian (LoG) filter. Particles were extracted in 256 × 256 pixel boxes scaled down by a factor of 2. An initial 2D classification step was used to remove incorrectly picked particles and subsequent 2D classification produced the 2D classes shown in the main text.

A β-gal model from the PDB (6CVM), low-pass filtered to 60 Å, was used as an initial model for 3D classification. Three-dimensional density maps were obtained from the native ES-IBD and control EER data set, using a subset of 16,400 and 5,100 particles, respectively, selected after multiple classifications. Particles were re-extracted in 560 × 560 pixel boxes and downscaled to 256 × 256 pixel boxes. The best 3D class from the last classification was used as a reference map, and scaled accordingly using the relion_image_handler program. The final structures were then produced using RELION’s automated refinement. Videos of the resulting 3D EM density maps were generated using ChimeraX (67).

## Supplementary Material

pgac153_Supplemental_Files

## Data Availability

The data presented here does not comprise any new structures or high-resolution structures. Thus, they have not been uploaded to a repository. However, all generated and analyzed data sets are available from the corresponding author on reasonable request.

## Previous presentation

These results were previously presented at Isolated Biomolecules and Biomolecular Interactions, 28 February 2022, and the Faraday discussions on challenges in biological cryo-electron microscopy, 14 July 2022.

## Preprints

A preprint of this article is published at https://doi.org/10.1101/2021.10.18.464782.
