# Supplementary material for: Mass-selective and ice-free electron cryomicroscopy protein sample preparation via native electrospray ion-beam deposition
Source: PNAS Nexus. 2022 Aug 6;1(4):pgac153. doi: 10.1093/pnasnexus/pgac153 (PMC9802471; doi:10.1093/pnasnexus/pgac153)
Supplement: pgac153_Supplemental_Files [file pgac153_supplemental_files.zip › PNASNEXUS-PNASNEXUS-2022-00533-T-s01.pdf]

SUPPLEMENTARY INFORMATION

Mass-selective and ice-free cryo-EM protein sample preparation via native electrospray ion-beam deposition

Authors

Tim K. Esser,[a,†] Jan Böhning,[b] Paul Fremdling,[a] Mark T. Agasid,[a,‡] Adam Costin,[b,$] Kyle Fort,[c] Albert Konijnenberg,[d] Joshua D. Gilbert,[e] Alan Bahm,[e] Alexander Makarov,[c,f] Carol V. Robinson,[a] Justin L. P. Benesch,[a] Lindsay Baker,[g,§] Tanmay A.M. Bharat,[b,h] Joseph Gault,[a,‡] Stephan Rauschenbach[a,i,*]

Affiliations

[a]Department of Chemistry, University of Oxford, Mansfield Road, Oxford, OX1 3TA, UK, [b]Sir William Dunn School of Pathology, University of Oxford, South Parks Road, Oxford, OX1 3RE UK, [c]Thermo Fisher Scientific, Hanna-Kunath-Straße 11, Bremen, 28199, Germany, [d]Thermo Fisher Scientific, Zwaanstraat 31G/H, Eindhoven, 5651 CA, The Netherlands, [e]Thermo Fisher Scientific, 5350 NE Dawson Creek Drive, Hillsboro, 97124, OR, USA, [f]Biomolecular Mass Spectrometry and Proteomics, Bijvoet Center for Biomolecular Research and Utrecht Institute for Pharmaceutical Sciences, University of Utrecht, Padualaan 8, Utrecht, 3584 CH, The Netherlands, [g]Division of Structural Biology, University of Oxford, Roosevelt Drive, Oxford, OX3 7BN, UK, [h]Structural Studies Division, MRC Laboratory of Molecular Biology, Francis Crick Avenue, Cambridge, CB2 0QH, UK [i]Max Planck Institute for Solid State Research, Heisenbergstrasse 1, Stuttgart, DE-70569, Germany, [†]current address: Thermo Fisher Scientific, 1 Boundary Park, Hemel Hempstead, HP2 7GE, UK, [‡]current address: Vertex Pharmaceuticals, 86-88 Jubilee Avenue, Milton Park, Abingdon, OX14 4RW, UK, [$]current address: Sydney Microscopy and Microanalysis, University of Sydney, Sydney, NSW 2006, Australia, [§]current address: Department of Biochemistry, University of Oxford, Oxford, OX1 3QZ, UK

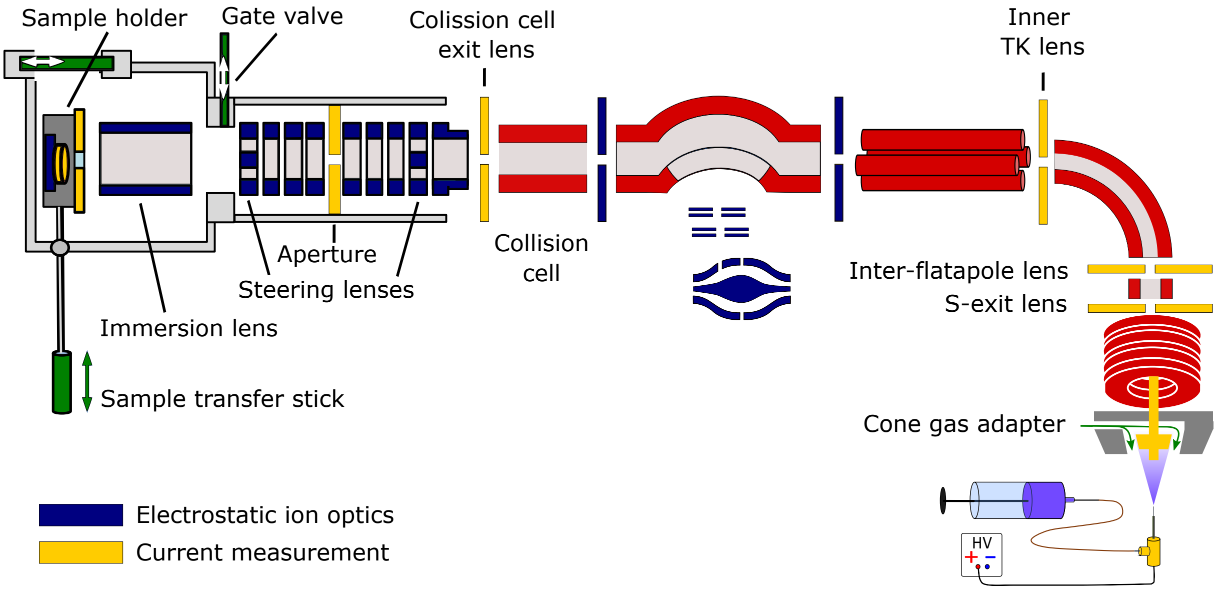

Figure S1: Schematic of the ES-IBD deposition instrument, consisting of a commercial, high-resolution mass spectrometry platform (Thermo Scientific Q Exactive UHMR instrument, right side) and home-made landing stage and sample holder (left side). The instrument combines RF (red) and DC (blue) ion optics. DC ion optics color-coded in yellow are connected to picoammeters (Model 9103, RBD Instruments), to allow for current measurement to optimize ion-beam transmission. The standard transfer capillary with an i.d. of 0.58 mm was exchanged for a custom capillary (based on 590129, CS Chromatographie) with an i.d. of 0.75 mm, which moderately increased transmission. The aperture of the S-exit lens was increased from 1.4 to 2.5 mm, increasing transmission up to tenfold. An additional pump (XDS35, Edwards) was installed as a dedicated backing pump for the source turbomolecular pump to account for the additional gas load. Ions are thermalized in the collision cell at a pressure of approx. 10^{-2} mbar. A beam collector (electrometer), downstream of the collision-cell exit-lens, was removed to allow for continuous transmission of the ion beam into the landing stage at a pressure of approx. 10^{-6} mbar. The latter allows the beam to be focused and steered onto the sample holder using only DC ion-optics. The sample holder comprises a retarding-grid ion-current detector, which records the ion-beam intensity, total beam energy, and beam energy distribution, as well as two sample positions with variable DC potential, which allows the landing energy to be controlled and to monitor the total charge deposited. Figure adopted from Fremdling et al..

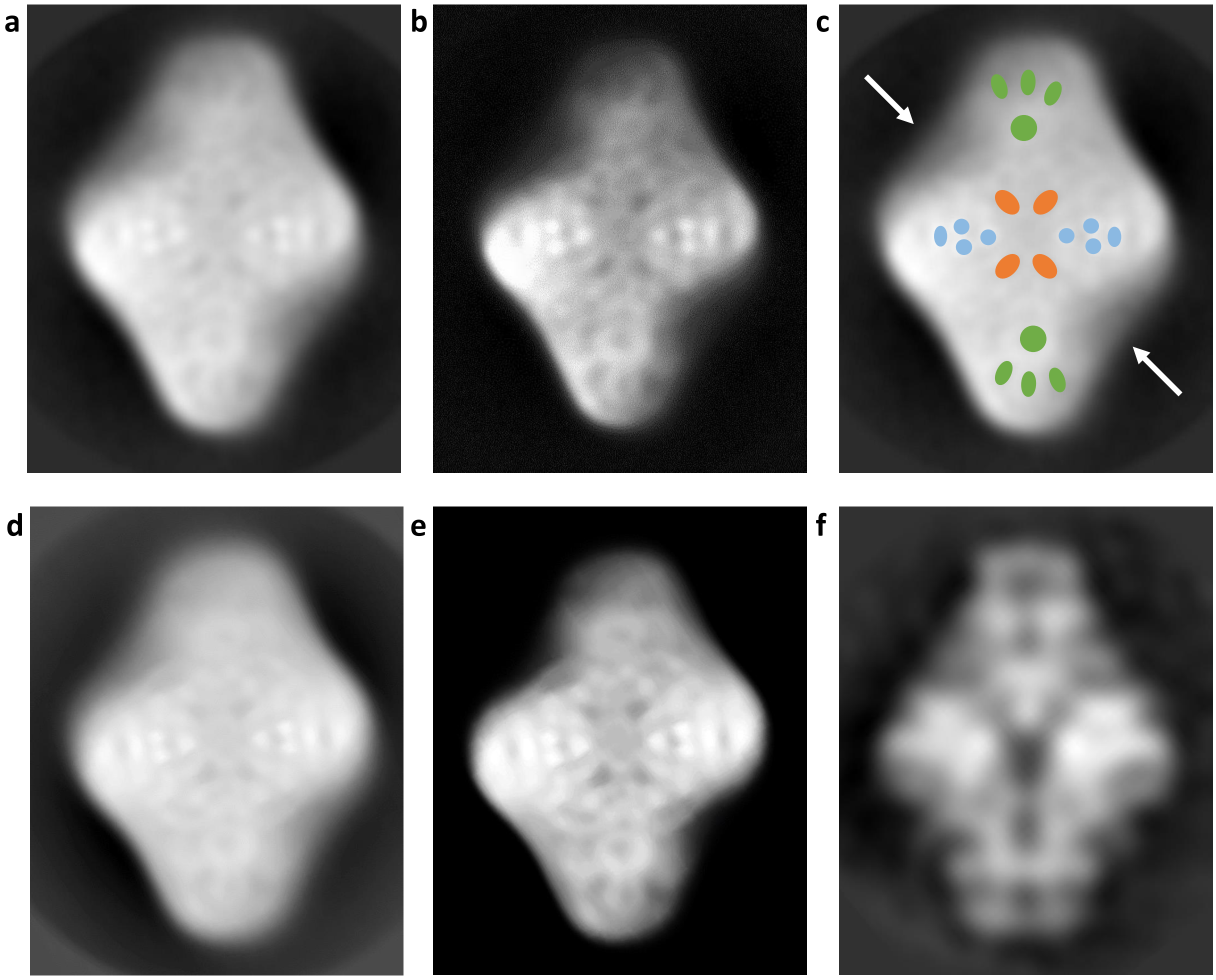

Figure S2: **a** Detailed view of the diamond-shaped class from the ice-free ES-IBD β-gal sample. **b** Same view with increased contrast and reduced brightness to highlight characteristic internal features. **c** Same view with marked features, including eight characteristic density maxima (blue), four minima around the center (orange), and eight minima at the tips (green). Arrows indicate edges with lower contrast, possibly due to direct interaction of the two corresponding subunits with the substrate. **d** and **e** are equivalent to **a** and **b**, except that the sample was plunged into liquid ethane instead of liquid nitrogen. The change of cryogen has no significant effect on sample quality. All features agree qualitatively with the class in **f**, obtained from the RELION 3.0 tutorial data set. However, the features in the class from the ES-IBD samples appear smaller and more diffuse, indicating a finite degree of variation in secondary and tertiary structure.

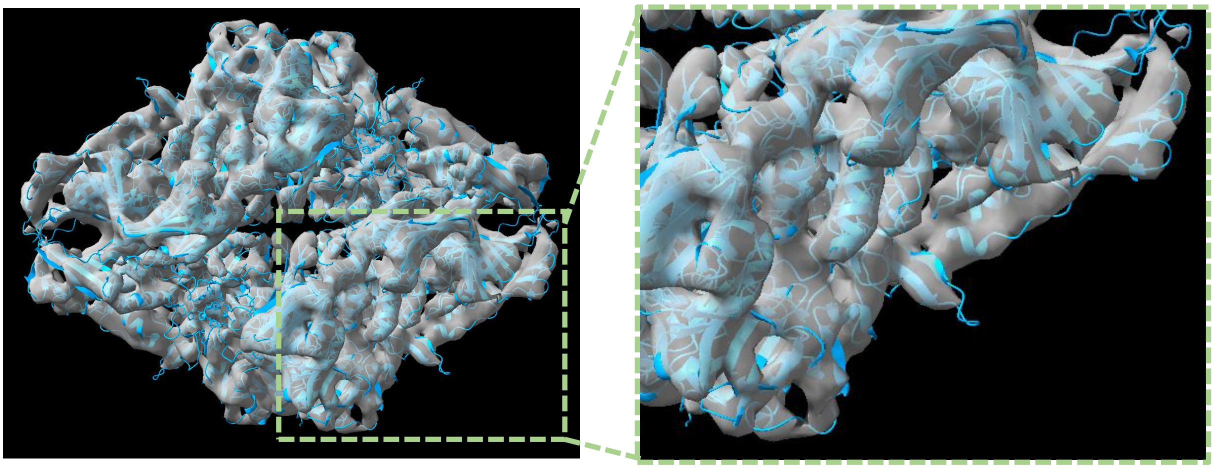

Figure S3: 3D structure from β-gal control sample in ice, obtained using the same solution as for the native ES-IBD samples (200mM ammonium acetate pH 6.9). The structure converged at a Gold-standard resolution (0.143 FSC) of 9 Å. The superimposed PDB model indicates very good agreement of secondary structure, allowing us to exclude protein preparation for mass spectrometry as a major bottleneck of the native ES-IBD workflow.

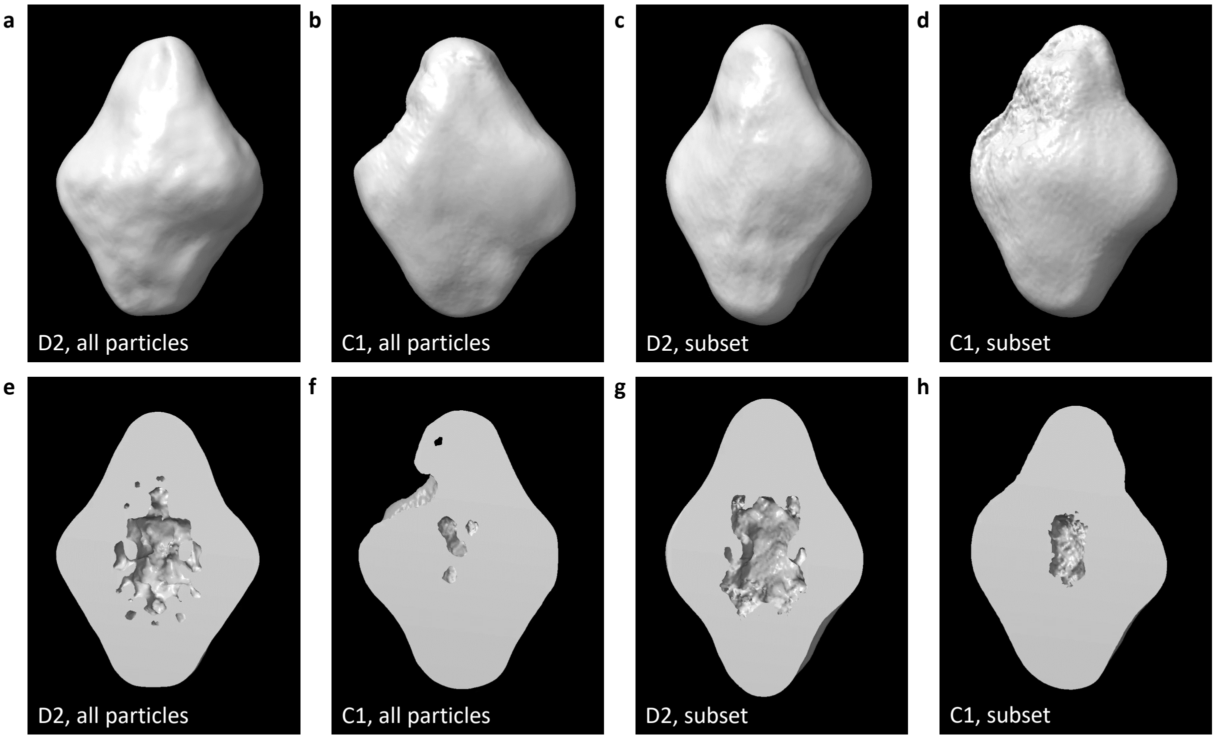

Figure S4: 3D EM density maps of the native ES-IBD sample of β-gal. The panels indicate if no symmetry (C1) or dihedral symmetry (D2) was applied during the 3D auto-refine step in RELION. Panels **a** and **b** show structures that were obtained using all 50,000 particles while panels **c** and **d** were generated using a subset of 16,400 particles. The panels in the second row show cross-sections of the structures above. See movie 1 for rotating structures.

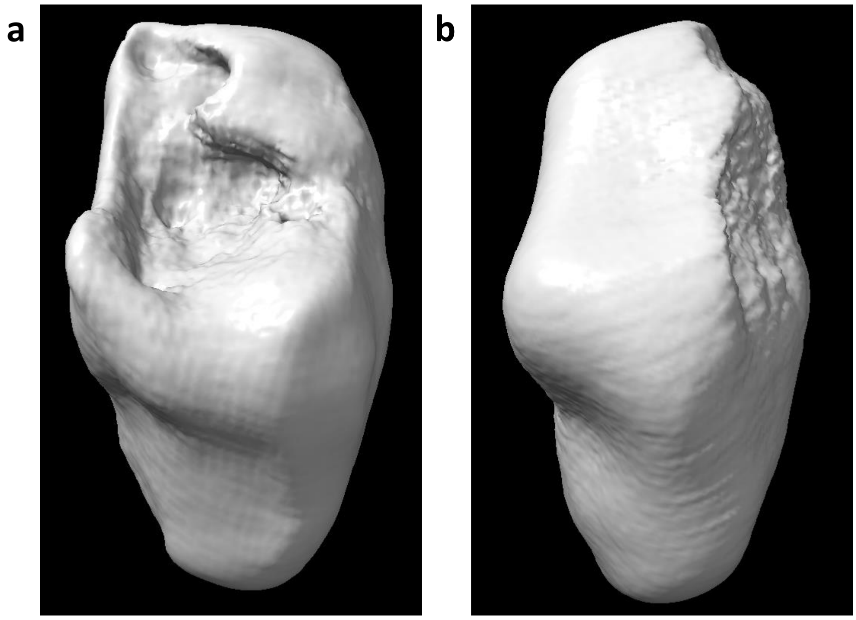

Figure S5: 3D EM density maps for ice-free ES-IBD β-gal sample obtained using C1 symmetry in RELION's automated refinement. **a** Map obtained using a subset of 50,000 particles showing localized deformation, possibly due to orientation dependent deformation on landing. **b** Map obtained from a subset of 16,400 particles, after multiple 2D and 3D classification steps, showing significantly less deformation.

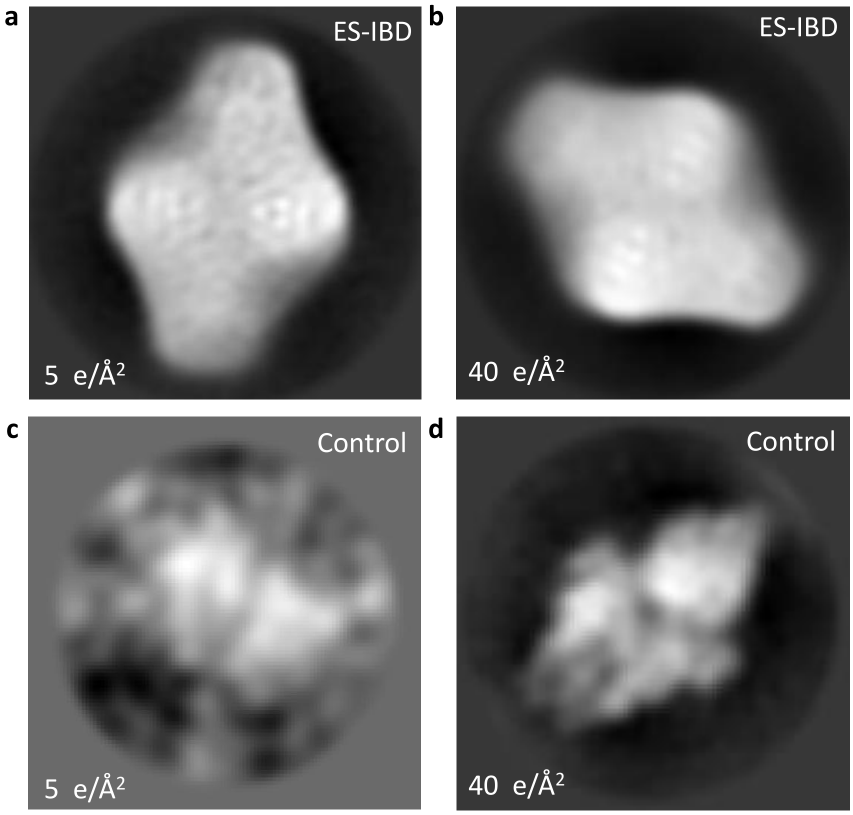

Figure S6: Dose dependence of 2D classes of β-gal for the native ES-IBD and control samples. Panels **a** and **b** show a 5 $e/\text{Å}^2$ and 40 $e/\text{Å}^2$ class, based on 10670 particles from the native ES-IBD sample. Panels **c** and **d** show a 5 $e/\text{Å}^2$ and 40 $e/\text{Å}^2$ class, based on 1349 particles from the control sample. The different doses were obtained by using a corresponding subset or all movie frames in the motion correction step in RELION. In both cases particles were selected from the 40 $e/\text{Å}^2$ data, and corresponding coordinates were used to extract the same particles from the 5 $e/\text{Å}^2$ data. The contrast of the control sample at low dose was not sufficient for successful alignment into a 2D class. This was possible however for the native ES-IBD sample, suggesting that lower doses are required when imaging ice-free samples, which can in principle reduce radiation damage. However, we cannot currently quantify or isolate the effect of radiation damage from that of the other factors discussed in the main text.

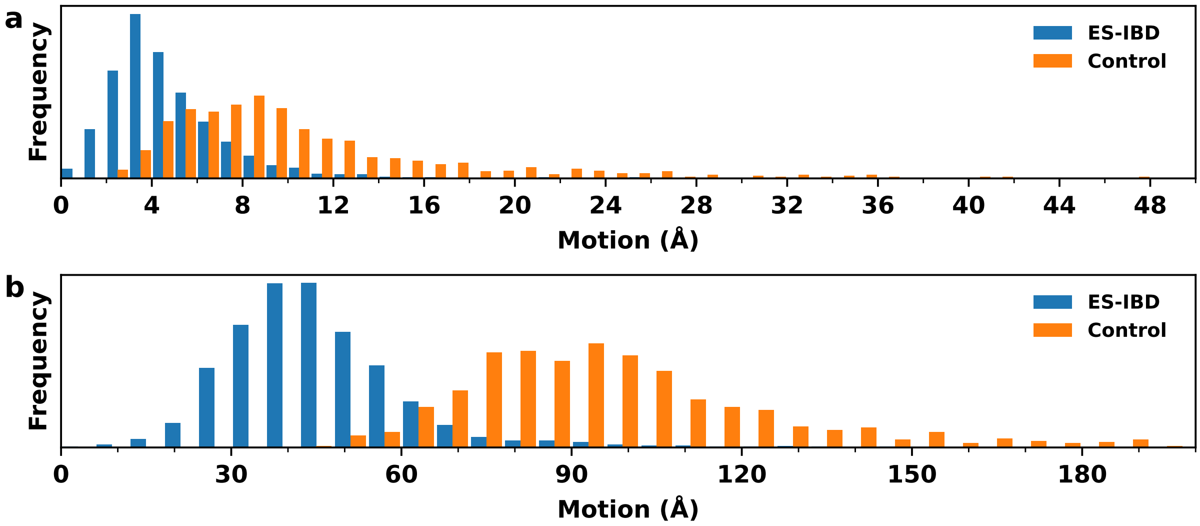

Figure S7: **a** Early (exposure $< 4\ e/\text{Å}^2$) and **b** total (full exposure of $40\ e/\text{Å}^2$) beam-induced motion for the ice-free native ES-IBD and control sample of β-gal, as given by RELION 3.1. Both early and late motion are about half for the ice-free sample, which seems plausible as there is no ice that can trap mechanical stress while the grid is cooled. While this is promising, further experiments are needed to unambiguously identify the respective contributions of the ice and the substrate.

References

[1] Fremdling, P.; Esser, T. K.; Saha, B.; Makarov, A.; Fort, K.; Reinhardt-Szyba, M.; Gault, J.; Rauschenbach, S. A Preparative Mass Spectrometer to Deposit Intact Large Native Protein Complexes. *arXiv:2203.04671 [q-bio]* **2022**, *preprint*.
